# Ontologies and Data Management: A Brief Survey

**DOI:** 10.1007/s13218-020-00686-3

**Published:** 2020-08-13

**Authors:** Thomas Schneider, Mantas Šimkus

**Affiliations:** 1 University of Bremen, Bremen, Germany; 2 TU Wien, Vienna, Austria

## Abstract

Information systems have to deal with an increasing amount of data that is heterogeneous, unstructured, or incomplete. In order to align and complete data, systems may rely on taxonomies and background knowledge that are provided in the form of an ontology. This survey gives an overview of research work on the use of ontologies for accessing incomplete and/or heterogeneous data.

## Introduction

In the digital age, we are dealing with a huge and growing amount of data in research, medicine, business and further areas. Information systems help us process and interpret that data. This is a challenging task because data used for a single purpose is often coming from heterogeneous sources, and is often unstructured and incomplete. In order to deal with these problems, *ontologies* provide taxonomies and background knowledge, which is useful (not only) for completion and alignment of data. In this survey we provide an overview of research on the use of ontologies for accessing incomplete and/or heterogeneous data. Since it is impossible to give a complete and detailed account of this broad and highly active research field, this overview treats most aspects briefly and omits others completely. In particular, we will focus on a family of ontology languages based on the so-called *Description Logics*, and only touch on other languages.

This survey begins with a discussion of ontology languages and available reasoning systems (Sect. 2). The main part of the survey (Sect. 3) treats the variety of research topics revolving around ontologies and data, divided into core topics (Sect. 3.1), extensions (Sect. 3.2), design-phase considerations (Sect. 3.3), and further topics (Sect. 3.4). We also give a brief overview of available resources (Sect. 4) and finish with a conclusion and a list of research challenges (Sect. 5).

## Ontologies

Ontologies originate from the philosophical branch of metaphysics, which studies existence. Since the 1970s, they have been used in the *Knowledge Representation (KR)* subfield of *Artificial Intelligence (AI)* for modeling the knowledge about various domains of interest, including (bio-)medicine, software engineering, cultural heritage, business processes, multimedia annotation, and the semantic web [142, 363]. Using an ontology, information systems have access to the represented knowledge and, via automated reasoning, can compute inferences. Early KR systems date back to the 1980s and early 1990s [83, 286, 288, 298, 323]. Thomas Gruber defined the notion of an ontology in the context of computer science as an “explicit specification of a conceptualization”, which is not limited to a taxonomy or a set of (conservative) definitions, but may also contain knowledge about the world [190].

In the following, we provide a brief account of available ontology languages and systems. A complete review of these topics would certainly require a separate survey article; hence we do not aim for completeness. Further information is provided in several books and book chapters [30, 33, 129, 363].

### Ontology Languages

Researchers have proposed ontology languages based on various formalisms. Frame-based languages such as F-Logic [11] are probably the earliest examples; they rely on the KR mechanism of *frames* [290], and their semantics is specified only operationally. In contrast, logic-based languages employ the formal semantics of their underlying logic, usually a suitable *Description Logic (DL)* or *first-order logic (FO)*. FO-based ontology languages, such as *Common Logic* [359] and the *Knowledge Interchange Format (KIF)* [169] feature all or most of the high expressive power of FO, but provide limited support for automated reasoning, given the undecidability of the basic reasoning problems in FO.

DLs comprise a prominent family of KR languages almost all of which are technically fragments of FO, although they use a different, variable-free syntax, tailored to KR. Unlike FO, DLs are typically decidable. We refer the reader to [30, 33] for details on the foundations of DLs. The various members of the DL family have been designed to provide a balance between expressive power and computational properties of the relevant reasoning problems that is suitable for various applications. Despite their restricted expressivity, DLs are powerful enough to formalize in a logic-based language (fragments of) popular modeling languages, such as UML and ER diagrams [13, 58]. Originating in frame-based languages, DLs have been studied since the 1980s, and this field is still active, as witnessed by the dedicated series of DL workshops.^1^

Among DL-based ontology languages, the Web Ontology Language OWL [211] stands out, being a W3C standard.^2^ A large number of ontologies is nowadays written in OWL, as witnessed by large corpora such as the NCBO BioPortal repository with over 860 bio-medical ontologies^3^ [345], the ontology repository at the University of Oxford with over 780 ontologies,^4^ or the OWL corpus at the University of Manchester with over 4500 ontologies^5^ [287].

The current standard OWL 2 is based on an expressive DL called $\mathcal {SROIQ}$ [208] and supports additional features such as datatypes [292] and a “safe” form of key constraints [322]. OWL 2 also provides the profiles EL, QL, and RL, which are considered *lightweight* ontology languages particularly suited to certain application areas. OWL 2 EL is based on the ${\mathcal {EL}}$ family of DLs and ensures efficient standard reasoning (e.g., classification) in polynomial time [29] via consequence-based reasoning algorithms. OWL 2 EL is used in healthcare and life-science ontologies, including SNOMED CT ontology (Systematized Nomenclature of Medicine—Clinical Terms), which systematizes the meaning of over 400,000 medical terms and is used in the healthcare systems of the US, the UK, and other countries [362]. OWL 2 QL is based on the *DL-Lite* family of DLs and enables very efficient sound and complete query answering using standard relational database technology [14]. We further discuss DL-Lite in Sect. 3.1. See also [38] for an introduction to lightweight DLs.

Inspired by database tradition, at least three families of logic-based ontology languages with features of relational databases have been developed to varying extents. The first family is based on *existential rules*, also known as *tuple-generating dependencies (TGDs)* [93, 297]. These languages embed into the well-understood Horn fragment of FO and conveniently generalize lightweight DLs such as those underlying OWL 2 EL and QL. They have been designed particularly towards enabling effective and efficient query answering. Among the three families, this one is most covered and best understood in the literature. We will discuss them in Sect. 3.2, together with further rule-based languages.

The second family comprises various extensions of DLs that support (legacy) database features, such as DLs with identification constraints and (path-)functional dependencies [100, 289, 334, 376], and variants of lightweight DLs with the bag (i.e., multiset) semantics [302].

The third family is in a much more experimental stage than the previous families, and consists of attributed DLs [250, 314]. Those have been designed to represent and reason with meta-knowledge, in the presence of knowledge graphs such as Wikidata [380] and DBpedia [256]. Attributed DLs are based on lightweight as well as expressive DLs.

Given the many reasons for using logic-based, and especially DL-based, ontology languages as well as the vast amount of available literature and ontologies, the remainder of this survey focuses almost exclusively on ontologies based on DLs.

### Ontology Systems

Developing, maintaining, and using ontologies requires tools such as editors, APIs and reasoners; the W3C website provides a brief overview of available implementations.^6^ Modern reasoners are typically based on sound and complete techniques for solving standard reasoning problems such as consistency checking, satisfiability testing, or classification, but there are also tools that solve more advanced relevant reasoning tasks such as query answering, module extraction, forgetting, explanation generation, abduction, etc.

The most common methods for solving standard reasoning problems include tableaux [207], hypertableaux [295], resolution [291], and consequence-based techniques. The latter were originally developed for Horn DLs such as tractable members of the ${\mathcal {EL}}$ family, resulting in highly efficient reasoning procedures based on completion rules [29]. The completion-based approach later became known as *consequence-based reasoning* and was carried over to more expressive Horn and even non-Horn DLs [53, 224]. In general, the technique of consequence-based reasoning combines ideas from hypertableaux and resolution. See also a recent survey [131].

A large number of DL reasoners are based on successful implementations and optimizations of these methods: (hyper)tableau-based reasoners such as FaCT++ [378], Pellet [358], HermiT [173], or Konclude [366] typically cover all of OWL 2. For OWL 2 EL, highly efficient consequence-based reasoners exist, such as CEL [37] or ELK [225]. Consequence-based reasoners for more expressive DLs include CB [224] and Sequoia [132].

We also note that there are hybrid reasoners that combine several other reasoners for more efficiency, sometimes relying on module extraction. Examples include Chainsaw [379], MORe [336], and PAGOdA [391].

For more information, see the proceedings of the ORE workshop^7^ and the list of reasoners maintained by the Information Management Group at the University of Manchester (last updated in June 2018).^8^

## Ontologies and Data

### Core Topics

We now discuss some of the basic topics related to the use of DLs to build ontologies, and employ such ontologies in the context of data management.

*Basic reasoning problems* DLs allow to model the domain of interest using only unary and binary predicate symbols, called *concept names* and *role names*, respectively. Different DLs support different constructors for building complex *concepts* and *roles*, and for expressing relationships among them. A *knowledge base (KB)* expressed in a DL is usually a pair ${\mathcal {K}}= ({\mathcal {T}},{\mathcal {A}})$, where ${\mathcal {T}}$ is a set of *inclusion axioms* (called *TBox*), and ${\mathcal {A}}$ is a set of facts (called *ABox*). In particular, ABoxes consist of *concept membership assertions* and *role membership assertions*. In the DL literature, the term “ontology” has at least two common usages: often it is used to refer to a whole KB ${\mathcal {K}}$ as above (this is especially true in the context of OWL ontologies), or to refer to the TBox of a KB. Intuitively, in the latter case, a DL KB consists of an ontology that is paired with some *data* in the form ABox assertions. Given the nature of this survey, we adopt this usage and use the term “ontology” (instead of “TBox”) to refer to the set ${\mathcal {T}}$ of inclusion axioms in a KB ${\mathcal {K}}= ({\mathcal {T}},{\mathcal {A}})$. In terms of classical logic, inclusion axioms can be seen as a special kind of *closed formulas* in first-order logic, and concept and role membership assertions are just a kind of *ground atomic formulas.*^9^ Sometimes it is convenient to think of an ABox simply as a collection of unary and binary (database) relations.

For an example, consider a KB ${\mathcal {K}}=({\mathcal {T}},{\mathcal {A}})$, where the ontology ${\mathcal {T}}$ contains the following inclusion axioms (all supported, e.g., in the basic DL $\mathcal {ALC}$):

$$\begin{aligned} \mathsf {ExchangeStudent}&\sqsubseteq \mathsf {Student} \\ \mathsf {Student}&\sqsubseteq \mathsf {Person} \\ \mathsf {Student}&\sqsubseteq \exists {\mathsf {attends}}.{\mathsf {Course}} \end{aligned}$$

The above ontology tells us that exchange students are students, that students are persons, and that a student must attend at least one course. Let the ABox ${\mathcal {A}}$ consist of the following assertions, whose meaning is obvious:

$$\begin{aligned} \begin{array}{rl} \mathsf {Student}(\mathrm {John}) &{}\quad \mathsf {worksIn}(\mathrm {John},\mathrm {CS}) \\ \mathsf {ExchangeStudent}(\mathrm {Ann}) &{}\quad \mathsf {worksIn}(\mathrm {Ann},\mathrm {CS}) \\ \mathsf {attends}(\mathrm {John},\mathrm {Logic}) &{}\quad \mathsf {worksIn}(\mathrm {Bob},\mathrm {CS}) \\ \mathsf {attends}(\mathrm {Bob},\mathrm {Logic}) &{}\quad \\ \end{array} \end{aligned}$$

The most basic reasoning task in DLs is *satisfiability testing*, that is, checking the existence of a *model* of a plain ontology ${\mathcal {T}}$, or a fully-fledged KB ${\mathcal {K}}= ({\mathcal {T}},{\mathcal {A}})$. Roughly speaking, a *model* ${\mathcal {I}}$ of ${\mathcal {T}}$ is a (possibly infinite) relational structure that satisfies all the inclusion axioms (i.e., all closed formulas) in ${\mathcal {T}}$. Furthermore, the structure ${\mathcal {I}}$ is a *model* of the full KB ${\mathcal {K}}$ if it additionally agrees with all the facts in ${\mathcal {A}}$. We remark that ${\mathcal {T}}$ or ${\mathcal {K}}$ may have (possibly infinitely) many models, or no model at all. Another standard reasoning task is *subsumption testing*, which consists of deciding whether one concept is at least as general as another concept, possibly in the context of other ontological axioms. This corresponds to checking whether an inclusion $C\sqsubseteq D$ seen as a logic sentence is valid, or is a logical consequence of some ontology ${\mathcal {T}}$. For example, $\mathsf {ExchangeStudent} \sqsubseteq \mathsf {Person}$ is a logical consequence of the above ontology ${\mathcal {T}}$, while $\mathsf {Person} \sqsubseteq \exists {\mathsf {attends}}.{\mathsf {Course}}$ is not. Another important task, which already points to data management, is answering *instance queries*. It consists of retrieving, given an ontology and an ABox, all *instances* of a given concept or role, i.e., all objects that can be inferred to belong to a certain concept, or pairs of objects connected by a role.

Most DLs can be seen as fragments of ${\mathcal {C}}^2$, the two-variable fragment of first-order logic with counting quantifiers [71, 374]. Unlike in full first-order logic, satisfiability testing is decidable in ${\mathcal {C}}^2$ [188, 319, 329]. The computational complexity of reasoning in DLs is a central topic of investigation in the DL literature, and it is well understood for most of the common DLs and for a wide variety of reasoning tasks, ranging from satisfiability testing to query answering. In fact, DLs vary greatly in terms of expressiveness and complexity of reasoning; e.g., lightweight DLs of the DL-Lite and ${\mathcal {EL}}$ families provide a limited set of constructors and thus are usually tractable. More expressive DLs like $\mathcal {SROIQ}$ [208] are not tractable.

**Fig. 1:**
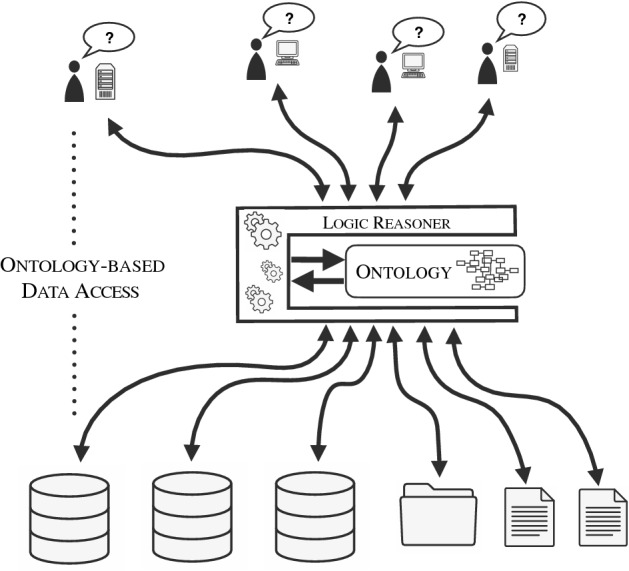
OBDA in a nutshell: a logic reasoner equipped with an ontology mediates access by multiple applications to multiple, possibly heterogeneous data sources

*Ontology-based data access* A major application of DLs is in *Ontology-based Data Access (OBDA)* [328] (see also [385] for a dedicated survey). This data integration paradigm aims at providing seamless access to multiple, possibly heterogeneous data sources. In OBDA, an ontology expressed in a DL provides a *conceptual view* of the application domain, while *mappings* relate the various data sources to the terms in the ontology. A user of the system poses a query using the vocabulary of the ontology, i.e., over the conceptual view. The OBDA system is then tasked to answer the user query by incorporating the information from the various information sources, possibly employing the domain knowledge in the ontology to infer new information. See Fig. 1 for an overview of OBDA.

For understanding the computational aspects and expressiveness of OBDA, the notion of an *ontology-mediated query (OMQ)* is very convenient. An OMQ is usually given as a pair $Q=({\mathcal {T}},{\mathcal {Q}})$, where ${\mathcal {T}}$ is an ontology, and ${\mathcal {Q}}$ is a *(user) query*. Intuitively, while ${\mathcal {T}}$ is meant to be constructed and maintained by domain experts, ${\mathcal {Q}}$ is an information request that can be made by a user of an OBDA system, who need not be a domain expert. Usually, standard database query languages, e.g., *conjunctive queries (CQs)*, are used to express user queries. In the spirit of classical database queries, OMQs are evaluated over ABoxes (recall that ABoxes can be seen as relational databases). The evaluation of an OMQ *Q* over an ABox ${\mathcal {A}}$ yields a relation, which is called the *query answer (to* *Q* *over* ${\mathcal {A}}$*)*. Roughly speaking, the answer to $Q=({\mathcal {T}},{\mathcal {Q}})$ over an ABox ${\mathcal {A}}$ contains a tuple $\mathbf {t}$  if and only if $\mathbf {t}$  is in the answer to the query ${\mathcal {Q}}$ in all models ${\mathcal {I}}$ of the KB ${\mathcal {K}} = ({\mathcal {T}},{\mathcal {A}})$. This is an example of the *certain answer semantics*, which is well known in databases.

#### Example 1

Consider an OMQ $Q=({\mathcal {T}},{\mathcal {Q}})$, where the ontology ${\mathcal {T}}$ is defined above, and the query ${\mathcal {Q}}$ is a CQ written as follows:

1 $$\begin{aligned} {\mathsf {q}}(x) \leftarrow \mathsf {Person}(x), \mathsf {attends}(x,y),\mathsf {worksIn}(x,\mathrm {CS}) \end{aligned}$$

The query ${\mathcal {Q}}$ asks for all persons who attend something and are members of the Computer Science department. We note the expression (1) is a simple example of a *rule*: the atom ${\mathsf {q}}(x)$ is called the *head atom*, while the atoms on the right-hand side of “$\leftarrow$” are called the *body atoms* of this rule. We will discuss more general rules later.

Consider the ABox ${\mathcal {A}}$ from above. Recall that our example ontology says that exchange students are a kind of students, that students are persons, and that every student must attend some course. Computing the certain answer to $Q=({\mathcal {T}},{\mathcal {Q}})$ over ${\mathcal {A}}$ yields precisely the *constants* (or, *individuals*) $\mathrm {John}$ and $\mathrm {Ann}$. The certain presence of $\mathrm {John}$ in the answer is due to ${\mathcal {A}}$ and the second inclusion in ${\mathcal {T}}$, which tells us that $\mathrm {John}$ is a person (slightly more precisely, $\mathrm {John}$ is a person in any model of ${\mathcal {K}}=({\mathcal {T}},{\mathcal {A}})$). In particular, if we evaluate ${\mathcal {Q}}$ in any model ${\mathcal {I}}$ of ${\mathcal {K}}=({\mathcal {T}},{\mathcal {A}})$, $\mathrm {John}$ is present in the query answer. The same is true for $\mathrm {Ann}$, but now all three inclusions of ${\mathcal {T}}$ play a role: the first one tells us that $\mathrm {Ann}$ is a student, and consequently from the second and the third inclusions we know that in any model of ${\mathcal {K}}=({\mathcal {T}},{\mathcal {A}})$ we have that $\mathrm {Ann}$ is person who attends some course. The individual $\mathrm {Bob}$ is not in the certain answer to *Q* over ${\mathcal {A}}$ because from the given assertions and inclusion axioms we cannot infer that $\mathrm {Bob}$ is actually a person. $\square$

The computational aspects of answering OMQs is a popular research topic, covering a range of DLs and (user) query languages. For instance, lightweight DLs of the DL-Lite and $\mathcal {EL}$ families support OMQ answering that is computationally not significantly more expensive than queries in the standard relational database setting (see, e.g., [14, 239]). However, answering OMQs based on CQs and expressive DLs is significantly more expensive in the worst-case (under standard assumptions in complexity theory), already because core tasks like satisfiability are ExpTime-hard for the basic expressive DLs like $\mathcal {ALC}$ [349]. ExpTime-completeness of answering OMQs based on CQs for $\mathcal {ALC}$ ontologies was established in [272, 312]. The problem is 2ExpTime-complete for extensions of $\mathcal {ALC}$ with inverse roles [272], with nominals [299], with role hierarchies and transitivity [152], or for positive first-order queries in plain $\mathcal {ALC}$ [311]. Algorithms for these problems have been obtained using a variety of techniques, e.g., reductions to consistency testing [175, 272], modified tableaux algorithms [307], resolution [214, 326], techniques based on tree automata [106, 309], type elimination [152, 153], and even combinations of some of the mentioned techniques [391]. Many of the above papers deal with computational complexity measured in the combined size of all input components (i.e., the size of the OMQ and the input ABox); they deal with the *combined complexity* of a problem at hand. However, motivated by the database perspective, the *data complexity* of query answering (i.e., the complexity measured in the size of the input ABox only, disregarding the size of the OMQ) has also received significant attention (see, e.g., [98, 213, 307]). For most common DLs, the data complexity ranges from very low (in particular, membership in AC$^0$ for DL-Lite) to coNP-completeness for expressive DLs (see Table 3 in [310]). Unfortunately, for the very expressive DLs that feature inverse roles, number restrictions, and nominals, in the best case decidability is established, but no tighter complexity results are available; this applies, e.g., to the DL $\mathcal {ALCOIQ}$ [346].

*Horn DLs* As mentioned above, expressive DLs suffer from intractable data complexity, which hinders their application in data management scenarios. This raised a natural question whether there exist useful fragments of expressive DLs like $\mathcal {SHIQ}$ that have tractable data complexity. The authors of [213] have identified *Horn-*$\mathcal {SHIQ}$ as one such DL, in which (the decision problem corresponding to) instance query answering is ${\textsc {PTime}}$-complete in data complexity. In [148] this tractability result was extended to CQs. Most DLs of the DL-Lite and $\mathcal {EL}$ families are considered to be *Horn DLs*, e.g., they are generalized by Horn-$\mathcal {SHIQ}$ [251]. The complexity of query answering in variants of $\mathcal {EL}$ was explored in [248, 252, 342], yielding complexity results that range from tractability to undecidability. The complexity of standard reasoning and conjunctive query answering in the expressive DLs Horn-$\mathcal {SHOIQ}$ and Horn-$\mathcal {SROIQ}$ has been studied in [308, 309], showing that these problems are ExpTime-complete in combined complexity, but are tractable in data complexity.

*Query rewriting* Database research is very mature, and a lot of highly optimized database systems exist and are used in almost any organization. For instance, the popular relational database management systems (DBMSs) provide efficient implementations for the SQL query language. CQs that are used in OBDA are closely related to *select-project-join* queries in SQL. Thus of course it makes sense to reuse existing DBMSs as much as possible, and the positive theoretic and practical results in this respect are one of the main reasons for the success of OBDA. Most OBDA systems—like Mastro [97] and Ontop [335]—use versions of DL-Lite as the ontology language. This lightweight DL has a very useful property, called *first-order rewritability (FO-rewritability)*. In particular, OMQs $Q=({\mathcal {T}},{\mathcal {Q}})$, where ${\mathcal {T}}$ is a DL-Lite ontology and ${\mathcal {Q}}$ is a CQ, can be translated into *unions of conjunctive queries* (UCQs), which are a mild generalization of CQs [14, 99]. That is, from *Q* we can obtain a UCQ $Q'$ such that evaluating $Q'$ over ${\mathcal {A}}$ (seen as a plain relational database) yields the answer to the OMQ *Q* over ${\mathcal {A}}$, for any possible input ABox ${\mathcal {A}}$. This shows very low data complexity (in particular, membership in AC$^0$), and opens the way for handling large amounts of data by reusing available database systems.

Unfortunately, the UCQ $Q'$ can easily become of exponential size in the size of the original OMQ *Q*. For this reason, the *succinctness* aspect of various rewritings for DL-Lite has received significant attention [64, 181, 186, 344]. E.g., the authors of [186] showed that polynomially sized non-recursive *Datalog* programs can be constructed for OMQs formulated using DL-Lite ontologies. Datalog is a standard rule-based language in (deductive) databases, with several efficient implementations. FO-rewritability for existential rules that often generalize members of the DL-Lite family have also received significant attention [42, 95, 127, 372].

Unfortunately, FO-rewritability is impossible for DLs whose data complexity (e.g., of consistency testing) is higher than AC$^0$, and this applies to most richer DLs discussed above. Fortunately, for many common DLs there exist rewritings into Datalog and its extensions, which have led to implemented tools (see, e.g., [154, 326, 377]). The seminal paper [214] showed that instance queries mediated by ontologies expressed in the DL $\mathcal {SHIQ}$ can be rewritten into *disjunctive* Datalog programs, which unfortunately could be of exponential size in the worst case. This rewriting is achieved by converting the input ontology into standard clauses in first-order logic, saturating the resulting theory using a resolution calculus, and then obtaining a disjunctive Datalog program by deleting all clauses with function symbols. Polynomial time rewritings into variants of Datalog were presented, e.g., in [5, 308]. The deep connections between OMQs and variants of Datalog (like *monadic* or *frontier-guarded* disjunctive Datalog) were also studied in [62].

*The combined approach* Query rewritings have been applied in several settings, from lightweight DLs to expressive DLs. The target languages change depending on the data complexity of the considered query answering problem. While for members of the DL-Lite family we usually have FO-rewritability, already for DLs as simple as $\mathcal {EL}$ FO-rewritability is impossible and we need a more expressive target language if we want to achieve rewritability. As an alternative to (pure) query rewritings, Lutz et al. introduced the *combined approach* to query answering in DLs, demonstrating the idea for the DL $\mathcal {EL}$ [280]. The idea is that the data is completed using the ontological knowledge only, in a query-independent way. This structure over-approximates query answers, i.e., it might provide answers that are not intended according to the certain answer semantics. Thus the next step is the filtration step that eliminates the non-answers from the candidate set. This approach was extended to rich fragments of DL-Lite in [238, 239, 276]. Lifting the combined approach to extensions of $\mathcal {EL}$ up to Horn-$\mathcal {ALCHOIQ}$ has been explored in [112, 160, 364]. Two techniques for eliminating unsound answers exist: query rewriting and reusing a database engine at hand [238, 239, 280], or implementing a separate post-processing procedure [276, 364].

*Navigational queries* Since in standard DLs we only use unary and binary predicate symbols, DLs are naturally related to graph-structured data and to graph databases. However, CQs discussed previously lack *navigational features* that are central in query languages for graph databases [50, 51, 128]. In particular, they lack constructs for the declarative specification of possibly complex *paths* that are to be traversed in a given structure, like a model of a DL ontology. Navigational queries like *regular path queries (RPQs)* in graph databases allow to specify complex paths by means of regular expressions built from roles [2, 104]. In recent years, several important generalizations of RPQs have emerged. For instance, *conjunctive regular path queries (CRPQs)* allow for flexible ways to specify joins of different RPQs [103, 109, 163], while *nested RPQs* are a close relative of the XPath query language for XML data [52]. These query languages are popular choices to access not only graph databases but also the graph-structured data on the Web, as they allow for recursion that is both computationally well-behaved and sufficient for expressing (variations of) reachability queries. The SPARQL 1.1 query language recommended by W3C as a standard for querying RDF data [381] includes the specification of *property paths*, which makes CRPQs one of the key building blocks for constructing queries in SPARQL 1.1.

Answering navigational queries mediated by DL ontologies has received significant attention in the literature. E.g., algorithms and complexity bounds for navigational queries in lightweight DLs have been studied in [61, 66, 245, 365]. Calvanese et al. have shown that CRPQ answering is 2ExpTime-complete for the very expressive DLs $\mathcal {ZIQ}$, $\mathcal {ZIO}$, and $\mathcal {ZOQ}$ [106, 107]. Recently this result was sharpened in [55], by showing that the complexity result holds even under the binary encoding of numbers, and by proving ExpTime-completeness of the query entailment problem for CRPQs with a bounded number of atoms. We note that $\mathcal {ZIQ}$, $\mathcal {ZIO}$, and $\mathcal {ZOQ}$ extend the more well-known DLs $\mathcal {ALCHIQ}$, $\mathcal {ALCHIO}$ and $\mathcal {ALCHOQ}$ with regular expressions as role constructors. Automata-based algorithms for CRPQs mediated by Horn-$\mathcal {SHOIQ}$ and Horn-$\mathcal {SROIQ}$ ontologies were proposed in [309], resulting in worst-case optimal ExpTime and 2ExpTime upper bounds, respectively. The work in [191] shows a 2ExpTime upper bound for CRPQs over $\mathcal {SQ}$ ontologies, where number restrictions are allowed both on transitive and non-transitive roles. We note that in some of the works above, generalizations of CRPQs to *positive regular path queries* are considered, and sometimes the ability to “walk” both along roles and role inverses is explicitly indicated by introducing *“2-way”* queries. Hence, the acronyms like *2RPQs*, *C2RPQs* or *P2RPQs* can be encountered in the literature.

*Inconsistency-tolerant query answering* In real-world data we often observe significant quality problems. In the presence of an ontology, some facts of a real-world ABox might easily become contradictory, which causes a logical inconsistency and renders query answering under the classical semantics meaningless. Several authors have considered *inconsistency-tolerant semantics* for query answering with DL ontologies, which are specifically geared towards inferring meaningful query answers in the presence of inconsistency. For more details on this important topic, we refer the reader to a dedicated survey included in this special issue [59].

*Closed-world and open-world assumptions* In the traditional database setting one makes the *closed-world assumption*. If a fact is not explicitly present in the database, then it is assumed to be false. This is not adequate for data integration scenarios, where information incompleteness naturally occurs. For this reason, standard OBDA settings drop this assumption, and make the so-called *open-world assumption* instead. It has been acknowledged however that that both of these assumptions are too strong, and that there is the need to explicitly control which parts of the knowledge should be considered complete and which could be seen as incomplete. E.g., in an OBDA system that integrates information about a city’s restaurants and bus routes, it may be useful to view the municipality-provided data about existing bus routes as complete, while a crowd-sourced collection of restaurants as incomplete. One way to achieve such control is via the so-called *closed predicates* (or *DBoxes*) [165, 278, 357]. In particular, we may know that the extensions of certain concepts and roles are *complete*, and thus should not vary in the different models of a given knowledge base. Unfortunately, reasoning in this mixed setting is computationally more challenging, e.g., the data complexity of query answering becomes NP-hard if closed predicates are added to (the core fragment of) DL-Lite [165]. Some ways to regain tractability in the presence of closed predicates in DL-Lite were presented in [278]. The recent work in [279] deals with more fine-grained notions of complexity as well as with the identification of first-order rewritable cases. The combined complexity of query answering in the presence of closed predicates was investigated in [299]. In general, the problem of combining the closed-world and the open-world assumptions has received significant attention. One can observe two lines of research here. First are the various combinations of DLs with *non-monotonic* rule-based languages, which we briefly discuss in Sect. 3.2. Another direction are the extensions of DLs with non-monotonic features (without a direct involvement of rules); see, e.g., [32, 69, 70, 82, 90, 91, 114, 115, 171, 187, 231, 249, 325, 331].

*Finite model reasoning* Most works on reasoning in DLs make the assumption that the domain of models may be infinite, and reasoning algorithms are often tailored to build finite representations of possibly infinite models. However, it has been acknowledged that in some data management applications one should only consider structures over finite domains. This is the case, e.g., when DLs are used to model relational databases. For instance, if we use a DL ontology to express some integrity constraints on a relational database, the models of the ontology should exactly be the finite databases that satisfy the constraints. This topic, known as *finite model reasoning*, has always been considered of interest in the DL community, but it is less developed than reasoning about possibly infinite models, mostly because it is significantly more challenging [217, 273, 343]. In this area *integer programming* has been very fruitfully applied [273, 329]. Another technique that has been studied for finite model reasoning in the so-called *Horn* DLs is the *cycle reversion technique*, which allows to reduce basic finite model reasoning problems to standard reasoning problems, i.e., reasoning over possibly infinite structures [217, 343]. We also recommend to see [161, 177, 179], where finite model reasoning for answering complex queries in the presence of ontologies in expressive languages is considered.

*Explanation services* In order for ontology-based systems to be truly useful to users, they must provide services to explain the outcomes of automated reasoning. For instance, if some inclusion is logically entailed by a possibly very large ontology, we may want to see which axioms of the ontology are actually responsible for the observed entailment. The task of computing such a *justification* is also known as *axiom pinpointing* and has received significant attention in the literature [39, 206, 222, 351]. We refer the reader to [324] for a dedicated survey.

The techniques developed for explaining entailments cannot be directly used for explaining *non-*entailments, such as missing answers to a query. That problem is addressed by *abductive reasoning*, which has also received significant attention, particularly the task of *ABox abduction* [138, 143, 156, 198, 226]: we are required to compute a collection of ABox assertions (an *explanation*) whose addition to the available knowledge base would imply some given fact (the *observation*). This task is non-trivial, not least because the space of possible explanations is infinite in general. The above ideas have also been applied in the context of query answering in DLs, where services are provided to explain why some tuple is or is not present in the answer of some OMQ [72, 111, 117, 118, 144].

*Evolving data* In most of the works discussed so far, data that is managed and queried with the help of ontologies is mostly assumed to be *static*. However, in most realistic scenarios data is not fixed, but it evolves because users are performing some data-manipulating actions, new information becomes available, some information becomes outdated, and so on. Reasoning about evolving data in the presence of ontologies is a core topic that is closely related to the classic AI area of reasoning about actions and change [333]. Many works in this classic area, especially the ones on decidable formalisms and actual systems, are in the realm of propositional theories. Reasoning about evolving data is more challenging, because the propositional setting is often not sufficient (e.g., it may require reasoning about a transition system with an infinite number of states). Reasoning about actions in DLs has received significant attention; see, e.g., [24, 36, 137, 260] for some positive decidability results. Further decidability and complexity results on reasoning (e.g., verification) in dynamic systems that involve databases or domain knowledge expressed using DLs can be found in [31, 35, 105, 108, 110, 201, 387]. This topic is closely related to the larger area of *temporal* DLs, which we will review in the next section.

### Extensions of Standard Ontology Languages

Even the most expressive DLs have a restricted ability to capture particular aspects of knowledge and/or data such as temporal changes, probabilistic behavior, or vagueness. In order to design ontology languages that overcome these limitations, several extensions of DLs have been studied, such as temporal, probabilistic, or fuzzy DLs, as well as generalizations of DLs with relations of higher arities, and combinations of DLs with rules.

*Temporal DLs* In many applications, data is changing over time, or the background knowledge has an inherently temporal component (e.g., in a medical context, one may think of patient records, or diseases and treatments having effects on a patient in the future). Standard ontology languages cannot capture temporal data or knowledge conveniently. For this reason, temporal extensions of ontology languages have been considered. *Temporal DLs (TDLs)* comprise a large and diverse family of DLs equipped with the ability to represent and reason over time. There are several ways to represent time: (1) in the point-based setting, discrete time points are assumed and operators from temporal logics such as LTL, CTL, and CTL* [157–159, 327] are used; (2) in the interval-based setting, time is represented by intervals and fragments of the Halpern-Shoham logic [200] are employed; (3) dense time is usually represented via variants of LTL that provide quantitative temporal operators [6, 7, 282]. Further ways to represent time include the use of datatypes [271] or formalisms in the spirit of action logics [15], but we focus on (1)–(3) here. Much of the work on TDLs since Schmiedel’s and Schild’s seminal papers [350, 352] is concerned with identifying combinations that provide a useful balance between expressive power on the one hand, and decidability and feasibility of the reasoning problems on the other hand. 

(1) *Point-based variants of TDLs* date back to Schild [350] and have been receiving particular attention in the past two decades, as witnessed by several surveys [16, 17, 20, 34, 167, 285]. Many point-based TDLs are fragments of 2-sorted FOL [375] and correspond to combinations of modal logics, such as fusions or products; the extensive study of many-dimensional modal logics [167] has had a significant impact on their study.

There are several degrees of freedom when combining a DL and a temporal logic; the respective design choices affect the expressive power and computational behavior, and sometimes drastically so. The design choices are of syntactic nature (such as the underlying DL and temporal logic, the amount of integration of the temporal operators) and semantic nature (whether to assume domains to be constant or varying over time, whether to admit time-invariant predicates, and more).

TDLs with *temporal concepts*—i.e., the use of temporal operators as concept constructors—are useful for referring to the temporal evolution of concepts (unary predicates). A “person who will be a student at some time in the future” is a possible temporal concept. TDLs with temporal concepts have been studied based on $\mathcal {ALC}$ and the temporal logics LTL and CTL. They provide only limited interaction between the DL and temporal components, and are computationally well-behaved, i.e., their standard reasoning problems—concept satisfiability, consistency—typically are not harder than in the component logics [18, 193, 285, 350, 384]. The interaction between both dimensions is increased significantly by adding *rigid roles*, i.e., the ability to assert that certain binary predicates are constant over time. This desirable modeling feature already makes combinations of LTL or CTL and the lightweight DL $\mathcal {EL}$ undecidable [21, 195], unless severe syntactic restrictions are imposed [196].

TDLs with *temporal roles* but without temporal concepts offer an “intermediate” amount of interaction between the dimensions and typically have decidable standard reasoning problems, with an increase in complexity compared to the component logics [23].

TDLs with *temporal axioms* allow for the use of temporal and Boolean operators on DL axioms (ontology or ABox or both), which again strongly limits the interaction between the components. For these TDLs, the standard reasoning problems as well as temporal ontology-mediated query answering has been studied extensively. The standard reasoning problems for the combination of $\mathcal {ALC}$ and LTL are not harder than the component logics in the case of, even when temporal concepts are added [31, 167, 285, 384]; decidability is preserved even when fixpoints are added [166] or the DL component becomes more expressive, e.g., $\mathcal {SHIQ}$ [285]. If the temporal component is replaced with a branching-time logic, then decidability is much harder (in the case of CTL) or undecidable (with CTL*) [54, 204]. Rigid roles lead to undecidability already in the presence of $\mathcal {EL}$, but not of DL-Lite [21].

For temporal ontology-mediated query answering (TOMQA), data and combined complexity are the most relevant complexity measures, and the notion of rewritability into standard query languages is strongly related. In this context, the data (ABox) is usually assumed to consist of time-stamped facts, the queries are equipped with temporal operators, and the background knowledge (ontology) is either considered static or uses temporal operators as well. Typically, constant domains and a linear flow of time (the naturals, the reals, or an interval thereof) is assumed.

For TOMQA with static ontologies, with LTL-based temporal CQs (TCQs), and with and without rigid symbols, data and combined complexity have been studied [27, 75, 76]. The ontology languages considered in these works range from lightweight to expressive DLs. We briefly summarize the results: for data complexity, tractability holds for ontologies in (a) “very light” DL-Lite dialects or (b) ${\mathcal {EL}}$ without rigid symbols; many cases that involve ontology languages up to $\mathcal {SHQ}$ are coNP-complete. In all cases, there is an ExpTime upper bound on data complexity. For the combined complexity, the results range from PSpace-completeness for the above cases (a) and (b) to 2ExpTime-completeness for the most expressive settings ($\mathcal {SHQ}$ with rigid symbols, or “heavier” DL-Lite dialects with role inclusions).

Rewritability has been investigated in the context of the *temporal database monitoring problem* [73], where a fixed temporal query is evaluated over temporal segments of a database. Three approaches have been developed [73]: rewritings into a temporal extension of SQL such as ATSQL [125], rewritings that only discard future operators from the query and allow the use of an algorithm based on *bounded history encoding* [124], and an extension of the latter algorithm that can handle future operators. An alternative approach to the monitoring problem consists in the *streaming data* scenario [315–317], leading to the Stream-Temporal Query Language STARQL [318], which again permits rewritings to SQL [360].

Temporal extensions of DL-Lite with temporal (LTL) concepts have been tailored carefully to ensure FO-rewritability of OMQs based on full two-sorted CQs or queries consisting of positive temporal concepts/roles [17, 19, 246]. A similar extension of $\mathcal {EL}$ ensures Datalog-rewritability for OMQs based on atomic queries [192].

TOMQA has also been studied for *clausal fragments of LTL*, where the ontology language is in the style of DL-Lite but additionally allows temporal operators in front of concept names, and the query language consists of atomic or positive temporal concepts, i.e., essentially LTL formulas without negation. Results on data complexity and rewritability into 2-sorted FOL and monadic second-order logic (MSO) have been obtained [19].

(2) *Interval-based TDLs* have also been considered for TOMQA. Decidable fragments of the Halpern-Shoham interval temporal logic $\mathcal {HS}$ [88, 89, 200] were combined with DL-Lite for answering atomic OMQs 22]; this setting was extended to CQs and fragments of multidimensional $\mathcal {HS}$ combined with Datalog [240].

(3) *TDLs with dense time* provide the ability to express that events take place within certain time intervals. Metric TDLs have been studied under several of the above design choices, i.e., with and without temporal concepts, temporal axioms and interval-rigid concepts and roles; most combinations are decidable, although often of higher complexity [26, 194, 215]. TOMQA has been studied for Datalog combined with Metric Temporal Logic [6, 247] and atomic queries; in particular, the data complexity for the non-recursive fragment is AC$^0$ [84]. This TDL has been used in practice in the contexts of turbine monitoring and weather monitoring [84], and in a video search system for ballet learners [332]; an implementation and evaluation have been described [84, 85].

*DLs with uncertainty and vagueness* The necessity to represent uncertain or vague information has fostered the study of probabilistic, possibilistic, and fuzzy extensions of DLs.

We start with a brief overview of probabilistic logics. The literature on probabilistic first-order and probabilistic DLs distinguishes two types of probabilistic information to be represented [40, 199, 388]: (1) *statistical* information about the world, via asserting probabilities for a randomly chosen individual to belong to a class/property; (2) *epistemic* information, via asserting a degree of belief that a certain individual belongs to a class/property. Probabilistic extensions of DLs have been reviewed and categorized via the two types by Zese [388, Chapter 12]; we provide a summary:

*Type 1 only:*  A simple extension of $\mathcal {ALC}$ with probabilistic axioms has been devised and equipped with incomplete local inference rules [203, 218].

*Type 2 only:*  Prob-$\mathcal {ALC}$ [275] allows probabilistic concept expressions and assertions in the style of Halpern’s probabilistic first-order logic of type 2 [199]. PR-OWL [113] adds the first-order probabilistic logic MEBN [255] to OWL. $\mathcal {EL}^{++}$-LL [300] combines the DL underlying the OWL EL profile with probabilistic log-linear models; reasoning has been implemented in E-LOG [303]. Several probabilistic extensions of DLs of varying expressivity are based on, or translatable into Bayesian networks [136, 141, 233, 386]; Recently, for a Bayesian extension of DL [121] the reasoner BORN [119] was implemented and query answering was studied [120]. Ontology-based access to probabilistic data and ontological data exchange were investigated under various semantics and based on various DLs and existential rules [86, 162, 182, 220, 266, 267]. Combinations of DLs with probabilistic logic programs were introduced for query answering and learning [96, 388] (the latter with a reasoner TRILL) and representing ontology mappings [268].

*Type 1 and 2:*  P-$\mathcal {SHOQ}(D)$ and P-$\mathcal {SHIQ}(D)$ [172, 263, 265] are based on a semantics from probabilistic default reasoning; for P-$\mathcal {SROIQ}$, satisfiability and entailment checking are implemented in the reasoner PRONTO [229, 230]. cr$\mathcal {ALC}$ [130] admits reasoning by translations to relational Bayesian networks and has also been studied in the context of learning [270].

As an alternative to probabilistic DLs, an extension of $\mathcal {ALC}$ (and more expressive DLs) based on possibilistic logic has been devised and implemented in the PossDL reasoner [205, 330]. Possibilistic $\mathcal {ALC}$ allows the annotation of axioms with weights, which are interpreted as lower bounds of necessity degrees based on the fuzzy set semantics of possibilistic logic [145, 259].

Yet another approach to modeling vagueness and imprecision is taken by fuzzy extensions of DLs which are related to classical fuzzy logics [126, 197] and many-valued logics [176, 228, 262]. They can model vagueness and imprecision by adding new degrees of truth to the standard values “true” and “false”. There are several semantics for fuzzy logics; they all are based on (non-Boolean) functions interpreting the Boolean operators. Fuzzy logics thus enjoy “truth functionality” in contrast to probabilistic or possibilistic logics: the truth degree of a formula in fuzzy logic is uniquely determined by the truth degrees of its subformulas.

Fuzzy DLs have been studied based on several (expressive and inexpressive) DLs and under several fuzzy semantics. Standard reasoning tasks as well as conjunctive query answering have been considered and implemented in several reasoners and ontology editor plug-ins. We limit ourselves to mentioning that essentially every truly fuzzy DL that allows for expressing terminological cycles is undecidable, see the discussion in a recent project report [28]. For a concise overview of fuzzy DLs, we refer the interested reader to a recent survey article [74], which also contains information on diverse applications of fuzzy DLs.

*Higher-arity relations* Recall that—with just a few exceptions (see, e.g., [101])—DLs allow building ontologies using only unary and binary relation symbols. Recall also that most DLs can be seen as fragments of ${\mathcal {C}}^2$, which in turn is a decidable fragment of first-order logic with counting quantifiers. Another prominent decidable fragment of first-order logic is the *guarded fragment (GF)*, which also captures many DLs yet features relations with higher arities and allows for formulas with an arbitrary number of variables [9, 216]. An even more expressive yet decidable fragment is the *guarded negation fragment* [47], which combines the ideas behind GF and another fragment called the *unary negation fragment* [353]. Another decidable fragment, called the *triguarded fragment*, combines GF with the two-variable fragment of first-order logic [347] (see also [81, 223]).

The ideas behind GF have spurred efforts to develop new expressive ontology languages that support higher-arity relations, often resulting in formalisms that are orthogonal to DLs in terms of expressiveness. One prominent example is *guarded tuple-generating dependencies (TGDs)* (also known as *guarded existential rules*) [93]. Roughly speaking, an ontology here is a set of rules with possibly existentially quantified variables in head atoms. Decidability of reasoning is ensured by requiring TGDs to be *guarded*: each rule is required to have a body atom (a *guard*) that contains all universally quantified variables of the given rule. The authors of [93] also relaxed this condition to *weak guardedness*, which excludes from guarding the variables that can be safely assumed to range over a small collection of known values. The notion of *frontier-guarded rules*, which generalizes guarded rules, was proposed in [42, 43]. Frontier-guarded rules are defined as guarded rules except that a guard in a rule is not required to contain all of the rule’s universal variables, but must contain all universal variables that occur in the head of the rule.

Many of the above and other related languages have been studied in various aspects, including decidability, data and combined complexity, query answering, expressive power, rewritability, and others. Query answering has naturally received most attention; see, e.g., [41, 48, 80, 93–95, 221]. Rewritability of query answering—targeting first-order logic (FO-rewritability) or more expressive languages like variants of Datalog—is an important tool in many of these paper, but sometimes it is studied on its own right (see, e.g., [4, 46, 185]). The combined approach (to query answering), discussed in Sect. 3.1 for DLs, was explored for existential rules in [183, 373]. Reasoning in the presence of closed predicates, which we discussed previously, has also received some attention [56, 60]. Please also see [184, 296] for tutorials on existential rules.

*Ontologies and rules* We have seen that in the area of query answering in DLs, CQs provide a basic way to specify what needs to be retrieved from the available data while taking into account a given ontology. We have also mentioned that CQs are an example of simple rules (see Example 1). Naturally, many authors have considered the use of more complex rules and rule sets in combination with ontologies; see, e.g., [154, 189, 209, 210, 253, 254, 258, 294]. This is a challenging topic as here usually *recursion* is considered, which brings a lot of expressiveness yet can easily lead to undecidability if no syntactic restrictions on rules are applied. Several authors considered extending rules with *default negation* (or, *negation as failure*), which allows rules to infer new facts based on the absence of information. Since such facts can be retracted after new information becomes available, inference with such rules is *non-monotonic*, which brings additional challenges. Often the so-called *answer set* semantics and the *well-founded* semantics known from logic programming are used to handle ontologies equipped rules with default negation; see, e.g., [8, 44, 149, 155, 231, 232, 264, 269, 293, 339–341]. We note that in these and other related works the level of separation between the ontology and the rules varies significantly, and often rules are an integral part of the knowledge representation toolkit, rather than just being tools for specifying user queries.

### Design-Phase Considerations

Most of the works discussed so far aim at providing intelligent services to the *users* of an ontology-based system. However, the *development* of such systems also requires reasoning support, which is often challenging since we do not have concrete data available (such problems are often called *static analysis* problems). Reasoning support for the development and optimization of queries, for building correct and accurate ontologies, as well as for the development and debugging of mappings in OBDA systems have received attention in the literature. We next discuss some of the reasoning services related to queries and ontologies, which is a rather well-established area. The development of OBDA mappings has received less attention; we only point to [67, 257] for some examples.

*Query containment* The most prominent example of a static analysis problem is *query containment* [3], which is a key task in contexts like query optimization, information integration, knowledge base verification (see, e.g., [116, 258] and the references therein). Given a pair $Q_1,Q_2$ of database queries, the query containment problem is to decide whether all the tuples in the answer to $Q_1$ are included in the answer to $Q_2$, for *any* possible input database *D*. The complexity of query containment has been studied extensively in database theory, starting from the classical NP-completeness result for plain CQs [122]. In the context of ontologies, a more representative problem is that of *query containment under constraints*, where the containment of query answers is tested quantifying over all databases that satisfy a given set of constraints. E.g., this problem has been considered for various database integrity constraints [219], and for queries over XML documents under DTD constraints [116]. In the context of ontology-mediated queries, this problem corresponds to checking query containment in the models of a given ontology. In this setting, the initial results for (unions of) CQs were presented in [101, 212]. A tight 2ExpTime upper bound for (an extension of) CRPQs was obtained in [106], but under the restriction that inverse roles and role functionality are not present together as language features. Decidability and tight upper bounds in the case when both inverses and functionality are allowed were presented in [109].

A more general setting, where the containment is tested for a pair of OMQs with two possibly different ontologies, has been explored in [65]. The authors show that this richer setting remains decidable for many common DLs and query languages, but higher computational costs are incurred. E.g., containment of instance queries mediated by lightweight DLs of the DL-Lite and $\mathcal {EL}$ families easily becomes intractable or even ExpTime-hard. Several 2NExpTime-completeness results for OMQ containment in expressive DLs can be found in [79]. A notable exception to decidability results is the case when functionality of roles or number restrictions are supported by the DL, e.g., containment of instance queries mediated by $\mathcal {ALCF}$ ontologies is undecidable [65]. The query containment problem for some important Horn DLs was studied in [63]. This problem for ontologies expressed using existential rules was studied in [49].

*Query emptiness* Another important static analysis problem is deciding *query emptiness*, which was first studied for DLs in [261]. Roughly speaking, given an OMQ $Q=({\mathcal {T}},{\mathcal {Q}})$, we would like to know if the answer to *Q* over ${\mathcal {A}}$ is empty, for any ABox ${\mathcal {A}}$ that is consistent with ${\mathcal {T}}$. If that is the case, then *Q* is clearly erroneous and should be corrected. Often in OBDA applications the signature of input ABoxes is significantly smaller than the signature of the ontology. Thus it is more suitable to narrow down the quantification from all ABoxes to ABoxes constructed using a restricted set of relation symbols. However, this restricted quantification often causes intractability and even undecidability [25]. In fact, several intractability and undecidability results for query containment in [65] are inherited from the lower bounds for query non-emptiness in [25]. For navigational queries, the closest work is [57], which considers satisfiability of XPath queries with DTD constraints. The query emptiness problem plays an important role also in other applications of ontologies in data management; for example, it is used for ontology *focusing* algorithms in [178].

*Modularity* Modern ontologies can reach considerable sizes; SNOMED CT (mentioned in Sect. 2.1) and the NCI Thesaurus [180] are prominent examples with (much) more than 100,000 axioms. Large ontologies pose serious challenges not only to the best optimized reasoners, but to all constituents of the ontology development process, such as navigation, editing, comprehension, and debugging. Module extraction and modularization help alleviate these problems and support application scenarios such as reuse, collaborative development, or versioning; see also [320]. Modularity has been studied extensively both theoretically and practically; see, e.g., [369]. The studied approaches can be divided into *a-priori* and *a-posteriori* approaches.

A-priori approaches allow the developer to impose a modular structure on an ontology at the time of development. They comprise suitable extensions of standard ontology languages, such as package-based DLs [45], distributed DLs [356], and ${\mathcal {E}}$-connections [134].

A-posteriori approaches can be applied to a given monolithic ontology to either extract a single module, or decompose an ontology into several modules. The term “module” can be understood in a broad sense: often it is a subset of the given ontology with certain logical guarantees that ensure an encapsulation of knowledge; however, logical guarantees as well as the subset requirement are not postulated by all a-posteriori approaches.

Module extraction and decomposition approaches without logical guarantees are usually based on some form of syntactic traversal of an ontology’s class hierarchy or other representation [305, 354, 370]. In contrast, approaches with logical guarantees are based on the notion of a *conservative extension (CE)*, which ensures that a subset ${\mathcal {M}}$ of an ontology ${\mathcal {O}}$ encapsulates all the knowledge in ${\mathcal {O}}$ about a given signature (part of ${\mathcal {O}}$’s vocabulary) [170]. CEs come in several variants (deductive, query-based, model-theoretic) and are generalized by the notion of inseparability [236], see also a recent survey on CEs [77]. Since inseparability and CEs are very hard or even undecidable for most DLs [170, 281, 283], most logic-based a-posteriori module notions either apply to inexpressive DLs [168, 235, 241] or approximate minimal modules while guaranteeing conservativity [133, 304, 337]; the latter often provide further guarantees such as self-containment and depletingness [242]. Among those, locality-based modules (LBMs) [133] are particularly versatile, well-behaved [139, 348], and available in the OWL API^10^; they have recently been generalized via an approach based on Datalog reasoning [337].^11^ Other studies have investigated the size of modules depending on the chosen module notion [168, 304]; further implementations exist (such as AMEX, or in the CEL reasoner; see the links in the original articles).

Decomposition approaches with logical guarantees exist in the form of signature decomposition [234, 321] or “true” ontology decomposition [135, 140]. The former induce modules of the input ontology that are no longer subsets (and related to interpolants, see below) and which exist only under certain conditions, which limits the benefits of these approaches for practical applications. Of the latter two approaches, the atomic decomposition (AD) partitions an ontology into *atoms* that are connected via a logically meaningful dependency relation [140]. The AD is implemented in the OWL API tools.^12^

*Uniform interpolation and forgetting* As just indicated, interpolants are related to modules; in particular, uniform interpolants (UIs) can be regarded as (non-subset) modules with very similar logical guarantees. UIs have been studied widely for DLs of various expressivity [243, 244, 277, 284, 301, 371, 383]. The same holds for the dual notion of forgetting, which is important for applications that require information hiding [123, 150, 237, 382, 389, 390]; see also a recent survey in this journal [151].

### Further topics

In addition to the topics discussed so far, further topics are being studied. The work on these topics is more ongoing or of a more specific interest. Here we list a few examples and refrain from a detailed discussion.

- Learning (ontologies and queries): see the survey [313]
- Privacy management: see [102, 306, 367]
- Stream reasoning with ontologies and/or (temporal) rules: see [92, 318, 338]
- Abstraction refinement for ontology materialization: see [87, 174]
- DLs of context: see [68, 78, 227, 355]
- Interactive query formulation and answering: see [10, 12, 164, 360, 361]
- Relaxations of query answering semantics: see [146, 147]
- Ranking OMQ answers: see [368]

## Resources

There are recent and comprehensive textbooks and handbooks on ontologies [363], DLs [30, 33] and, more generally, on knowledge representation [202] and Semantic Web technologies [142]. The proceedings of the *Reasoning Web* summer schools provide tutorial notes introducing a wide variety of topics; see the DBLP entry.^13^ There is also a recent survey on OBDA [385], and a recent Festschrift [274] gives an overview on active research topics in DLs.

Pointers to various tools, such as reasoners, ontology repositories, and web applications, can be found on the OWL web page at the University of Manchester.^14^

A list of related events can be found in the editorial of this special issue.

## Conclusion and Challenges

In this survey we have provided a rather broad overview of DL research that either directly envisions the use of ontologies in data management, or plays some important supporting role in this vision. While many works have been visited here, this survey is not meant to provide a full picture, or a complete review of the available literature of the field. Our goal was to discuss some representative works so that a newcomer can use them to start exploring the vast body of related literature.

Despite the big research efforts of the last years, many challenges remain to be addressed in order to realize the potential of ontologies in data management. For example, we need methods and techniques that enable interactive query answering, context-aware data access, managing information change, managing streaming data, assessing data quality, data analytics, a finer analysis of computational complexity. The challenges are too many to be discussed here, but luckily they have been collected and discussed in detail in Section 6 of [385] and in Section 5 of [1], which we recommend to read.

## Footnotes

1 http://dl.kr.org/.

2 https://www.w3.org/TR/owl2-overview/.

3 https://bioportal.bioontology.org/.

4 http://www.cs.ox.ac.uk/isg/ontologies/.

5 http://owl.cs.manchester.ac.uk/owlcorpus.

6 https://www.w3.org/2001/sw/wiki/OWL/Implementations.

7 https://dblp.uni-trier.de/db/conf/ore/.

8 http://owl.cs.manchester.ac.uk/tools/list-of-reasoners.

9 We are making a simplifying assumption here. ABoxes, in general, are allowed to contain more sophisticated assertions, but this usually does not add expressiveness to the formalism at hand, and thus we ignore them for the sake of presentation.

10 https://github.com/owlcs/owlapi.

11 http://www.cs.ox.ac.uk/isg/tools/PrisM/.

12 https://github.com/owlcs/owlapitools/.

13 https://dblp.uni-trier.de/db/conf/rweb/.

14 http://owl.cs.manchester.ac.uk/tools/.
